# M’Clintock’s Introductory Lecture

**Published:** 1841-03

**Authors:** 


DR. M'CLINTOCK'S INTRODUCTORY LECTURE.

This lecture was delivered by Dr. M'Clintock in November last, at the opening of the winter course in the Philadelphia School of Anatomy, of which, we believe, he is the founder. In matter and manner the lecture is excellent. It treats in a sufficiently fervid style of the advantages of anatomical knowledge, but it contains not a word on that subject, the truth of which every scientific physician would not hastily subscribe. The following passages are specimens of the lecture, and convey useful truths to the younger members of the profession:

"Twenty-three hundred years ago, flourished HIPPOCRATES, the Father of Medical Science, who, like Homer and Raphael, in their respective arts, not only created this science, but brought it to a high degree of perfection, by the impulsive force of his genius and the unwearied energy of his research. So accurate were his experiments and so profound his observations, that he unfolded the history of acute diseases with so much clearness and precision, that twenty centuries have hardly detracted from the value, or added to the amount of his researches. But he far from attaining the same degree of perfection in Surgery. And why? Because the circumstances with which he was surrounded—the prejudices of an ignorant and superstitious people, and their religious veneration for the asylum of the departed—by depriving him of the opportunity of dissecting the human body, raised up insurmountable obstacles to the proper study of Anatomy; and his knowledge of the subject founded chiefly upon the external appearance of the body, and on the examination of the structure of those animals which were supposed to approach nearest to man, was necessarily limited and imperfect. In the study of acute diseases, commonly occurring, which presented strongly marked symptoms, and only required a just and sagacious observation of their succession and relations to each other, in order to the formation of a correct history of them, these imperfect notions were sufficient; but in surgery, the want of anatomy is immediately felt, and the consequence was that the science, even in the hands of so great a master, made little or no improvement. And it remained in its infancy, for nineteen hundred years longer, until, in the beginning of the 16th century, the genius and boldness of Vesalius gave new life to anatomy. The darkness that had so long hung over surgery was seen pierced by the new and brilliant light which the advancement of anatomy diffused over all branches of medicine, and under the illuminating power of this clear and certain flame, the art soon assumed a new aspect, and suddenly rose to a high pitch of perfection, in the hands of Ambrose Paré, the distinguished surgeon of Charles IX, whose great and well deserved reputation saved his life, in the memorable night of the massacre of St. Bartholomew. To this day, the countrymen of Paré claim for him the same high place among surgeons, that is assigned to Hippocrates among physicians. And among all the great names that adorn the history of Surgery in later times, the Petitis, the Desaults, the Le Drans, the Munrons, the Hunters, the Coopers and the Physicks,—you will not find one, the foundation of whose high renown was not deeply laid in the study of anatomy; here they prepared themselves for those successful operations which made them the admiration and wonder of their own times, and for those brilliant discoveries and inventions which have transmitted their glory undiminished to ours—nay, which shall crown every one of these names with the immortal reward of an imperishable fame.

"There is no quality more indispensable to the surgeon than that undisturbed coolness of mind which cannot exist without a strong and just confidence in his own powers, and in his preparation for the arduous task before him, when he is called upon to perform some important operation. This just self-confidence is essential to every pursuit in life. To quote the language of Bulwer, "if you rob Genius of its confidence, you clip the wings of the eagle." No great enterprise in the walks of science, literature, art or business, has ever been accomplished without it; it nerves the arm of the warrior, it gives energy and power to the decisions of the statesman, and it enables the scholar to struggle on, through years of unceasing labor, with full hope of the final reward of all his toils. But to the surgeon of all other men, is this calm confidence and self-reliance is indispensable. He is called to the side of a wounded man—life is fast ebbing away—his judgment must be almost as rapid as his eyesight, and his action must follow, as rapidly, the decisions of his judgment, or the patient will die in his hands. Now, under such circumstances, a man must either be thoroughly ignorant of anatomy, or fully learned in it, in order to have confidence in himself.

"But your attention should also be carefully directed to the necessity of examining the dead, for the purpose of ascertaining the seat and the effects of disease. It is very true that a great proportion of your practical skill in detecting the character of disease is to be obtained by patient investigation at the bed-side of the sick; but it is equally true, that without much time and labor bestowed on cadaveric inspection, you can never have that quick perception of the nature of internal maladies, that ready apprehension of the immediate seat of the disease, and that almost intuitive perception of the hidden workings of the morbific agency in the system, which ought to be exhibited by every feeble and inadequate ideas upon these subjects, and he who trusts to them, and refuses to store his mind with the results of accurate observation obtained in post mortem examinations, will always find his views of disease unsatisfactory to himself and his consequent practice doubtful and uncertain. And your desire to witness and take part in cadaveric inspections, should not be confined to those cases in which death has been the result of rare and singular diseases, but should be extended to those which may be regared as even of ordinary occnrrence. To quote the appropriate language of M. Hodgkin, "it is only by this practice that the student can learn to distinguish the almost endless varieties of aspect which parts and organs assume, and to avoid confounding three things essentially distinct,—healthy, diseased, and cadaveric appearances; and to him who is engaged in practice, it is no less necessary, in order to keep up his knowledge, and to rectify, confirm, and improve his diagnosis."

"To the members of the class, I will say, you will be expected to acquire most of your knowledgo here obtained, from actual dissections made with your own hands. This is the *only* way in which you can become expert practical anatomists; you may listen to lecture after lecture, you may read volume after volume, but if you do not unite with your hearing and reading, actual dissections of yonr own, your acquisitions will neither be accurate, solid or permanent. Accordingly, our rooms here are "dissecting rooms" rather than "lecture rooms." A school in which you can study for yourselves, as well as be instructed by another: the dead subject, in addition to the voice of the living instructor, is to reveal to you the secrets of anatomy." Y.

